# Coinfection of Pulmonary Blastomycosis and Tuberculosis in an Immunosuppressed Patient: A Challenging Clinical Case

**DOI:** 10.7759/cureus.71729

**Published:** 2024-10-17

**Authors:** Francisco de la Peña-Camacho, Hugo E González-Chávez, Karen S Arrazola, Emmanuel Reyes-Ferreira, Francisco J Lugo-Rincon Gallardo

**Affiliations:** 1 Internal Medicine, General Hospital of the Institute of Social Security and Services for State Workers, Queretaro, MEX

**Keywords:** blastomycosis, co-infection, immunosuppression, pulmonary infections, tuberculosis

## Abstract

Fungal and tuberculosis lung infections are considered differential diagnoses due to their clinical and radiological similarities, but they are not mutually exclusive. In rare cases, both entities may manifest in patients with immunosuppression, which is an indicator of high mortality. This is predominantly due to the low level of suspicion, which consequently results in a delay in treatment and thus represents a significant diagnostic challenge. This article describes the clinical evolution of an immunosuppressed patient with co-infection of pulmonary blastomycosis and tuberculosis to emphasize the importance of recognizing these associations in daily practice.

## Introduction

Blastomycosis is a rare pyogranulomatous infection that is exceptionally diagnosed in Latin America and develops as a result of inhaling *Blastomyces* conidia [1]. Similar to tuberculosis, it can be considered an opportunistic infection primarily affecting the lungs. In contrast, the latter is the infectious disease that causes the most deaths worldwide [2]. Although the correlation between tuberculosis and pulmonary fungal infections in immunosuppressed patients is well documented with overlapping symptoms primarily including cough, fever, night sweats, weight loss, and dyspnea; there is limited information regarding the association between these two entities, which are known to have a high mortality rate when presented with acute respiratory distress syndrome (ARDS) [3].

## Case presentation

**Figure 1:**
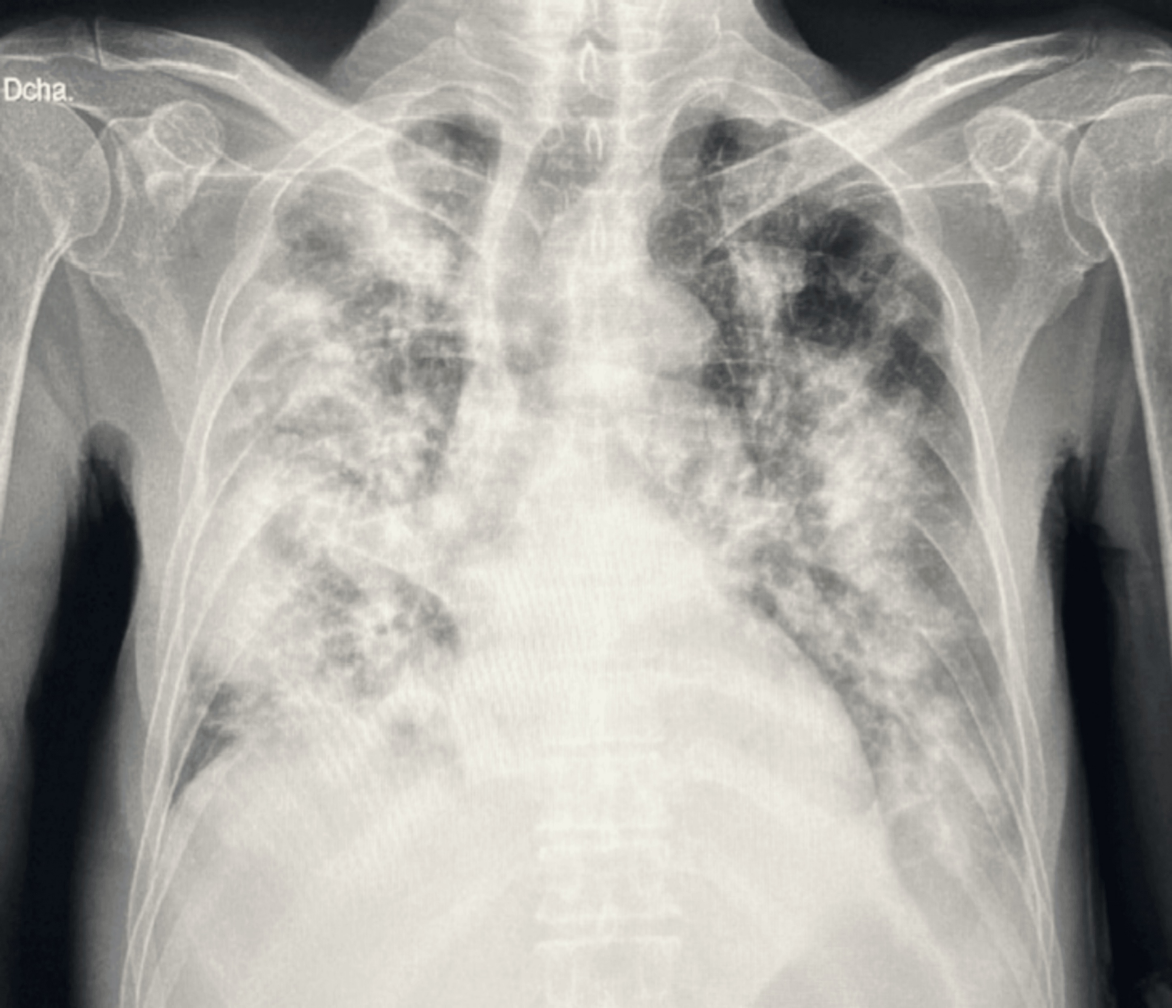
Portable chest X-ray Anteroposterior projection with multiple bilateral alveolar infiltrates, some with a nodular appearance.

**Figure 2:**
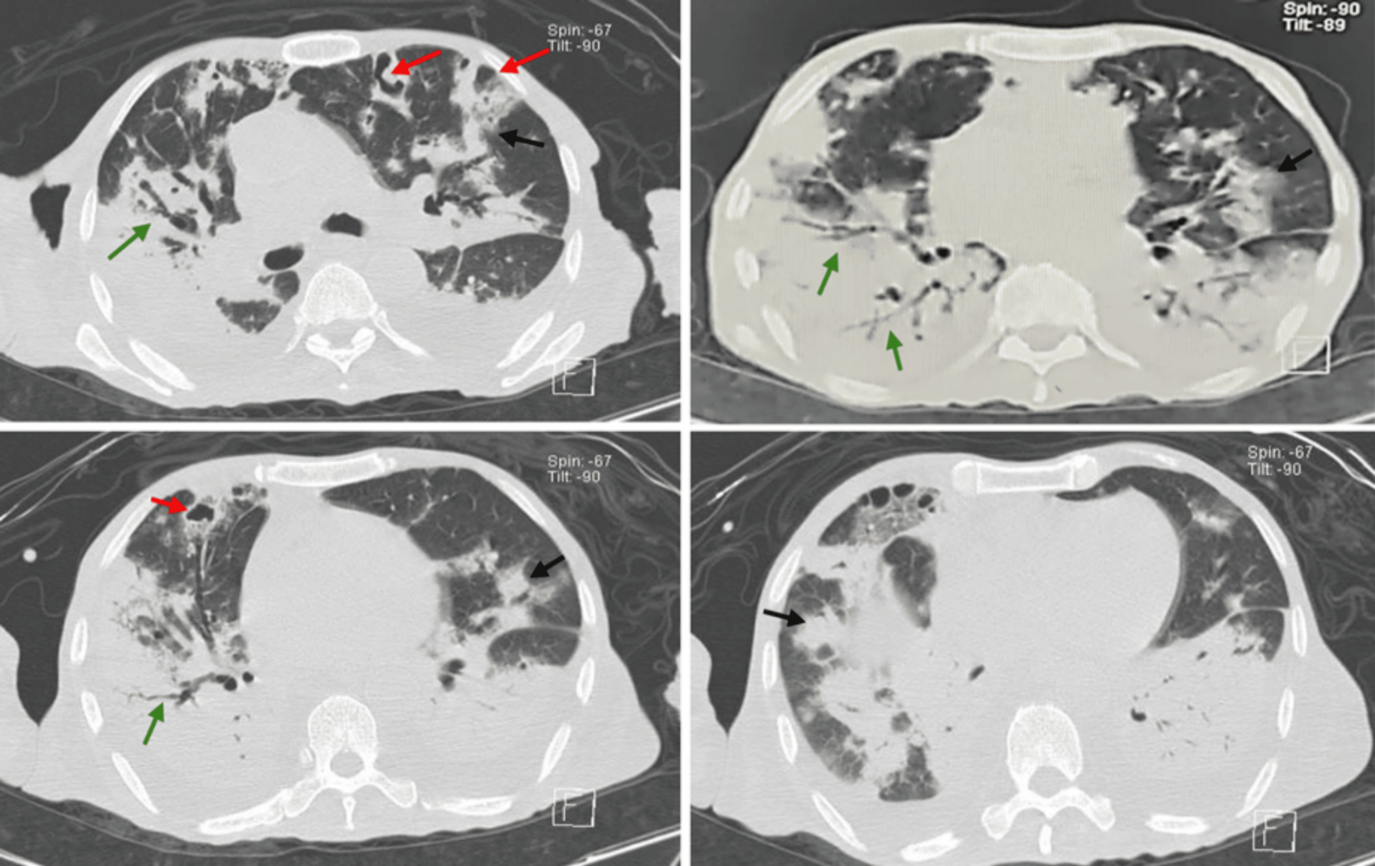
Plain Chest CT Scan Lung window, axial slices showing a cobblestone pattern (black arrows), varicose bronchiectasis (green arrows), and multiple cavitations (red arrows) in the medial segments.

A 71-year-old male presented to our hospital with a six-month history of asthenia, arthralgia, unintentional weight loss, and progressive hyporexia. These symptoms were accompanied by recurrent fever, progressive dyspnea, cough with yellowish sputum, hemoptysis, and desaturation in ambient air. His past medical history was significant for living in a rural area near waterways, with no history of Bacillus Calmette-Guérin (BCG) immunization, and three siblings who had died from tuberculosis. Additionally, he had a history of heavy smoking and alcohol use, along with self-medication with 10 to 30 mg of oral prednisone daily for approximately one year. Upon hospital admission, he exhibited a tendency toward tachycardia with a heart rate between 90-110 bpm, tachypnea with a respiratory rate between 20-24 breaths per minute, a temperature of 36°C, blood pressure of 106/75 mmHg, and desaturation in ambient air down to 80%, requiring supplemental oxygen up to 15 L/min. The physical examination revealed a cachectic patient with a body mass index of 14.58 kg/m², signs of dehydration, generalized weakness, tachypnea, and disseminated crackles predominantly on the right side during lung auscultation. Laboratory investigations indicated hemoglobin (Hb) 7.8 g/dl, mean corpuscular volume (MCV) 65 fl, mean corpuscular hemoglobin (MCH) 21 pg, platelets 67,000/uL, leukocytes 1,480/uL, neutrophils 1,360/uL, lymphocytes 800/uL, albumin 1.4 g/dl, urea 68 mg/dl, procalcitonin 0.37 ng/dl, C-reactive protein (CRP) 13 mg/dl, ferritin 2,228 ng/ml, and arterial blood gas with pH 7.53, partial pressure of carbon dioxide (pCO2) 38 mmHg, partial pressure of oxygen (PaO2)/fraction of inspired oxygen (FIO2) 94, and bicarbonate (HCO3) 31 mmol/L. The chest X-ray demonstrated diffuse radio-opacities (Figure 1), while the CT scan exhibited a cobblestone pattern, bronchiectasis, and multiple cavitations (Figure 2). Infectious causes were considered, and SARS-CoV-2 and influenza infections were excluded, the viral panel exhibited a negative result. The tumor markers were also negative (CA 19-9, Carcinoembryonic Antigen, Alpha-fetoprotein, CA-125, Prostate-Specific Antigen. This was part of the protocol to rule out malignant etiologies such as stomach, pancreatic, biliary tract, liver, prostate, and colorectal cancers). Sputum culture was positive for* Blastomyces sp.*, the galactomannan antigen test was negative, leading to the initiation of itraconazole treatment with an initial dose of 200 mg three times daily for three days, followed by a maintenance dose of 200 mg twice daily. Additionally, acid-fast bacilli were documented in sputum, necessitating the incorporation of anti-tuberculosis treatment (this was based on an intensive phase using rifampicin, pyrazinamide, ethambutol, and isoniazid in a combination of tablets at dosages of 150 mg, 75 mg, 400 mg, and 300 mg, respectively, with three weight-adjusted tablets administered every 24 hours from Monday to Saturday for 10 weeks, followed by a maintenance phase consisting of rifampicin and isoniazid tablets at dosages of 400 mg and 300 mg, with two tablets taken three times a week for four months).​​​​​​​ After an 18-day hospitalization, the patient was discharged due to clinical improvement, with supplemental oxygen support and a plan to complete antifungal treatment for 12 months and the six-month course of anti-tuberculosis treatment. Follow-up continued in outpatient consultations, showing good adherence to treatment, no adverse reactions, weight gain, and gradual social reintegration, with negative control sputum smears.​​​​​​​

## Discussion

Epidemiological data on pulmonary blastomycosis in Mexico and Latin America is limited, with the majority of documented cases occurring in North America [4], where the disease is associated with wooded areas and waterways [5]. Its chronic presentation can often be confused with tuberculosis or malignancy [6], making differential diagnosis and the documentation of such associations crucial. In contrast, tuberculosis is endemic in Mexico, with over 28,000 cases reported in 2022 [7]. Both pathogens exhibit opportunistic behavior, highlighting the importance of the patient's risk factors, such as steroid use, malnutrition, age, smoking, lack of BCG immunization, alcoholism, neutropenia, and leukopenia [6], all of which contribute to the severity of pulmonary disease. Imaging findings in both diseases are variable and may include massive lesions and cavitations [8]. The presence of ARDS is associated with a significant increase in mortality rates, ranging from 50% to 80%. In most cases, death occurs within the initial days of hospitalization [9]. The definitive diagnosis of blastomycosis is confirmed by the growth of the organism from a clinical sample. Unlike other mycoses, such as Candida and Aspergillus, contamination or colonization does not occur [5]. For tuberculosis, the detection of acid-fast bacilli by microscopy is the fastest and most economical diagnostic tool [10]. Amphotericin B and azoles represent the primary treatment for blastomycosis, with most patients showing improvement within the initial 14-day period [11].

## Conclusions

In immunosuppressed patients, the presence of opportunistic pulmonary infections, such as those observed in this case, is common. However, there are few reports associating pulmonary blastomycosis with tuberculosis due to the similarity in clinical presentation and the high mortality associated with both conditions. Early recognition is crucial for establishing appropriate and timely treatment to eradicate the infections and limit disease progression, thereby reducing the impact of potential sequelae and complications. It is important to note that for immunosuppressed patients with moderate to severe ARDS, the treatment of choice for blastomycosis is amphotericin B (Evidence level A-III). Unfortunately, this was not administered in this case due to hospital limitations. Nevertheless, the patient responded well to azoles, completing a 12-month regimen, suggesting that further studies and epidemiological research are needed.
